# Common molecular basis for MASH and hepatitis C revealed via systems biology approach

**DOI:** 10.3389/fonc.2024.1442221

**Published:** 2024-11-04

**Authors:** Yongwei Cheng, Zihao Song, Ye Liu, Xichao Xu, Dali Zhang, Yigui Zou, Liang Liu, Yinzhen Zeng, Wenwen Li, Daming Bai, Dongling Dai

**Affiliations:** ^1^ Key Laboratory for Precision Diagnosis and Treatment of Pediatric Digestive System Diseases, Endoscopy Center and Gastroenterology Department, Shenzhen Children’s Hospital, Shenzhen, China; ^2^ GMU-GIBH Joint School of Life Sciences, The Guangdong-Hong Kong-Macao Joint Laboratory for Cell Fate Regulation and Diseases, Guangzhou Medical University, Guangzhou, China; ^3^ School of Laboratory Medicine, Hubei University of Chinese Medicine, Wuhan, China

**Keywords:** metabolic dysfunction-associated steatohepatitis (MASH), hepatitis C, bioinformatics analysis, differentially expressed genes, Budesonide

## Abstract

**Background:**

Metabolic dysfunction-associated steatohepatitis (MASH) is characterized by liver inflammation and damage caused by a buildup of fat in the liver. Hepatitis C, caused by hepatitis C virus (HCV), is a disease that can lead to liver cirrhosis, liver cancer, and liver failure. MASH and hepatitis C are the common causes of liver cirrhosis and hepatocellular carcinoma. Several studies have shown that hepatic steatosis is also a common histological feature of liver in HCV infected patients. However, the common molecular basis for MASH and hepatitis C remains poorly understood.

**Methods:**

Firstly, differentially expressed genes (DEGs) for MASH and hepatitis C were extracted from the GSE89632, GSE164760 and GSE14323 datasets. Subsequently, the common DEGs shared among these datasets were determined using the Venn diagram. Next, a protein-protein interaction (PPI) network was constructed based on the common DEGs and the hub genes were extracted. Then, gene ontology (GO) and pathway analysis of the common DEGs were performed. Furthermore, transcription factors (TFs) and miRNAs regulatory networks were constructed, and drug candidates were identified. After the MASH and hepatitis C cell model was treated with predicted drug, the expression levels of the signature genes were measured by qRT-PCR and ELISA.

**Results:**

866 common DEGs were identified in MASH and hepatitis C. The GO analysis showed that the most significantly enriched biological process of the DEGs was the positive regulation of cytokine production. 10 hub genes, including STAT1, CCL2, ITGAM, PTPRC, CXCL9, IL15, SELL, VCAM1, TLR4 and CCL5, were selected from the PPI network. By constructing the TF-gene and miRNA-gene network, most prominent TFs and miRNAs were screened out. Potential drugs screening shows that Budesonide and Dinoprostone may benefit patients, and cellular experiments showed that Budesonide effectively inhibited the expression of genes related to glycolipid metabolism, fibrosis, and inflammatory factors.

**Conclusion:**

We extracted 10 hub genes between MASH and hepatitis C, and performed a series of analyses on the genes. Molecular docking and *in vitro* studies have revealed that Budesonide can effectively suppress the progression of MASH and hepatitis C. This study can provide novel insights into the potential drug targets and biomarkers for MASH and hepatitis C.

## 1 Introduction

Liver disease has emerged as a major cause of global health burden. It accounts for approximately 2 million deaths per year worldwide, including 1 million due to complications of cirrhosis and 1 million due to viral hepatitis and hepatocellular carcinoma (HCC) (1, 2). Among them, Metabolic dysfunction-associated steatohepatitis (MASH) and viral hepatitis are the main causes of liver cirrhosis and HCC (1).

Metabolic dysfunction-associated steatotic liver disease (MASLD) is a chronic condition marked by excessive fat deposition in hepatocytes, unlated to alcoholic consumption or other specific liver-damaging factors. It is usually accompanied by insulin resistance and diabetes mellitus. The prevalence of MASLD is 25% to 30% and continues to rise each year as high-fat diets become more prevalent (3). MASH is the progressive form of MASLD. In the development of MASLD, about 5% to 20% of patients progress from simple fatty liver disease to MASH, a more severe disease defined by macrovesicular steatosis, hepatocyte injury (ballooning), and liver inflammation (4). MASH may culminate in cirrhosis and HCC and is currently a leading cause of liver transplant (5).

Hepatitis C virus (HCV) is a hepatotropic RNA virus that can cause acute and chronic hepatitis, with progressive liver damage resulting in cirrhosis, decompensated liver disease, and HCC. In 2020, there were an estimated 56.8 million hepatitis C virus infections worldwide (HCV RNA viraemic prevalence 0.7%), China (9.48 million), Pakistan (7.39 million) and India (6.13 million) being the three countries with the highest disease burden (2, 6). The early stage of hepatitis C is often asymptomatic, with only 56% of patients being aware of their infection. Missing the opportunity for treatment can lead to more serious liver injury in the late stage. Compared with HBV, a greater proportion of hepatitis C progresses to cirrhosis and liver cancer.

Furthermore, indirect estimates from much research suggest that 3% to 6% of adults at the population level have MASH. With the worldwide epidemics of diabetes and obesity, the proportion of MASH patients is expected to increase over the next decade (7). Therefore, the probability for interaction between MASH and hepatitis C is significant. Several studies have shown that hepatic steatosis is also a common histological feature in HCV infected patients (8, 9). Analysis of the results of 25 studies, collectively including 6400 patients, showed that up to 55.54% of these HCV infected patients have variable degrees of hepatic steatosis (10). The average prevalence of HCV-associated MASH is between 4% and 10% (11). This is also supported by the observation that the degree of liver steatosis is directly related to the level of HCV replication, as measured by serum HCV RNA (8). Steatosis was significantly reduced in hepatitis C patients after antiviral therapy. Therefore, much more work needs to be done to explore the strict association between hepatitis C and MASH.

In this study, we first analyzed three microarray datasets downloaded from the Gene Expression Omnibus (GEO) platform to obtain differentially expressed genes (DEGs) for hepatitis C and MASH. Then we analyzed the identified common DEGs using Gene Ontology (GO) analysis, Kyoto Encyclopedia of Genes and Genomes (KEGG) analysis, and Protein-Protein Interaction (PPI) Networks analysis. In addition, transcription factors (TFs), potential target miRNA, and associated diseases of hub genes were predicted. Finally, we identified a number of compounds that may be used in the treatment of MASH and chronic hepatitis C based on hub genes. Our results support the possibility that MASH and hepatitis C share the same molecular basis and regulatory pathways, which might serve as potential therapeutic targets for patients with MASH and hepatitis C and facilitate the development of targeted drugs.

## 2 Materials and methods

### 2.1 Gene expression profile data collection

In this study, three gene expression datasets were collected from the Gene Expression Omnibus (GEO) database. GSE14323 was derived from a hepatitis C associated hepatocellular carcinoma study, GSE89632 and GSE164760 were derived from two studies of hepatocellular carcinoma in patients with non-alcoholic steatohepatitis. In all cohorts, no patients were diagnosed with both MASH and hepatitis C. Datasets are available at https://www.ncbi.nlm.nih.gov/geo/ for more information. Pertinent information for the selected GEO datasets used in this study was summarized in **Table 1**.

**Table 1:** Accession information for datasets downloaded from the GEO database.

Accession	GPL	Etiology	Sample size case/control	Country	Year
GSE14323	GPL571	HCV	**HCV: 96** (41 HCV-cirrhosis; 38 HCV-HCC; 17 HCV-cirrhotic tissues from HCC patients) **Healthy: 19**	USA	2018
GSE164760	GPL13667	MASH	**MASH: 156** (74 NASH, 29 adjacent non-tumor tissues; 53 tumor tissues from NASH-HCC patients) **Healthy: 6**	Spain	2021
GSE89632	GPL14951	MASH	**MASH: 19** **Healthy: 24**	Canada	2017

### 2.2 Identification of differentially expressed genes

Background expression value correction and data normalization were conducted for the raw data in each dataset using an R package (Affy; version 1.52.0). Probes in each data file were then annotated based on the appropriate platform annotation files. Probes without matching gene symbols were removed when a probe corresponded to more than one gene, the preceding gene was deleted. In instances where different probes mapped to the same gene, the mean value of all probes mapping to that gene was taken as the final expression value for that gene. Batch effects were removed using the R package (SVA; version 3.48.0) Then, the Linear Models for Microarray Analysis R package (limma; version 3.56.2) was applied for differential expression analysis. Those genes with a p-value < 0.05 and |log2FC| > 0.5 were deemed to be the DEGs. In addition, the overlapping DEGs between MASH and hepatitis C were delineated using the ggvenn R packages.

### 2.3 Gene ontology, pathway enrichment analyses, and gene set enrichment analysis

Gene Ontology (GO) is a commonly used bioinformatics tool that provides comprehensive information on gene function of individual genomic products based on defined features. This analysis consists of three facets: molecular functions (MF), biological processes (BP) and cellular components (CC). Enrichment analysis was performed using DEGs overlapping, and R package (clusterProfiler; version 4.8.1) was used in this analysis process. KEGG is considered as a knowledge base for systematic analysis of gene functions, linking genomic information with higher order functional information. For quantifying the top listed functional items and pathways, and a statistical threshold criterion with p-value < 0.05 and q-value < 0.05 were used to identify significant GO terms and KEGG pathways. Additionally, we performed GSEA analysis of GSE datasets by gseGO (R clusterProfiler package).

### 2.4 Protein–protein interaction network construction and hub gene analysis

In order to analyze the connections among the proteins encoded by identified DEGs, DEGs were uploaded to Search Tool for the Retrieval of Interacting Genes (STRING, https://string-db.org/), a database of known and predicted PPI networks. The results with a minimum interaction score of 0.4 were visualized in Cytoscape. And then, we used the Cytoscape plug-in Minimal Common Oncology Data Elements (MCODE, http://apps.cytoscape.org/apps/mcode) to screen out key protein expression molecules. Furthermore, CytoHubba, a Cytoscape plugin app, providing a user-friendly interface to explore important nodes in biological networks, was utilized with the maximal clique centrality (MCC) method to explore the PPI network for hub genes.

### 2.5 Construction of regulatory networks of transcription factors and miRNAs

To determine major transcriptional variations, we analyzed the interaction networks of hub genes with miRNAs and transcription factors (TFs) using the NetworkAnalyst platform. Specifically, the NetworkAnalyst platform was utilized to locate topologically credible TFs from the JASPAR database that tend to bind to the hub genes. For hub genes and miRNA network construction via NetworkAnalyst platform, the TarBase and miRTarBase databases were used to extracted miRNAs with hub genes focused on topological analysis.

### 2.6 Screening of potential therapeutic compounds

An online resource, Drug Signatures Database (DSigDB), connects drugs/compounds to their target genes. To study the drug molecular properties of MASH and hepatitis C, we used the DSigDB library under the Diseases/Drugs function in Enrichr (https://maayanlab.cloud/Enrichr/enrich).

### 2.7 Gene-disease association analysis

The DisGeNET database contains one of the most comprehensive collections of genes and variants associated with human disease. Based on hub genes, we identified diseases and chronic health problems using DisGeNET database under the Diseases/Drugs function in Enrichr.

### 2.8 Molecular docking

Molecular docking that an established in silico structure-based method is widely used in drug discovery. Docking enables the identification of novel compounds of therapeutic interest, predicting ligand-target interactions at a molecular level, or delineating structure-activity relationships (SAR), without knowing *a priori* the chemical structure of other target modulators. In our study, key targets of MASH and hepatitis C were obtained through hub genes identification, including CD4 and SRC. Next, the crystal structures of these key proteins were downloaded from the Protein Data Bank (https://www.rcsb.org/) for further molecular docking. The molecular structures of potential drug molecules were obtained from the ZINC (https://zinc.docking.org/) database. The Autodock tools (version 1.5.4) was utilized in all docking experiments, with the optimized model as the docking target. The screening method is restricted to molecular docking, and molecular dynamics simulation has not been carried. In addition, the results were shown with binding energy (BE), a weighted average of docking score, to assess the reliability and describe the accuracy of the ligand positioning. Pymol (PyMOL Molecular Visualization System 2020) was used for 3D visualization of the docking results.

### 2.9 Cell culture

The human hepatoma cell line Huh7.5 and HCV full-length genomic plasmid were kept in our laboratory. Huh7.5 cells were cultured in DMEM with 10% fetal bovine serum (FBS) at 37°C in a 5% CO_2_ humidified atmosphere. The cells were treated with 0.2 mM PA and 0.1 mM OA for 24 h to create a hepatocyte steatosis model *in vitro*. In the drug experiment, the Budesonide powder was first dissolved in Dimethyl sulfoxide (DMSO), then formulated into an aqueous solution, and 1 mM of the aqueous Budesonide solution was added to the cell culture media. As a control group, only the same dose of DMSO aqueous solution was added to the cell culture medium.

### 2.10 HCV transfection

Twenty-four hours before transfection, 4.2×10^5^ Huh7.5 cells per well were seeded in six-well plates. For transfections, 10 μg of HCV recombinant plasmid was linearized with XbaI, treated with mung bean nuclease, purified, and *in vitro* transcribed using T7 RNA polymerase (Promega) (100 μl total). The resulting HCV RNA transcripts were mixed with 150 μl Opti-MEM (Invitrogen) and incubated for 10 min at room temperature, mixed with 255 μl transfection complex [5 μl of Lipofectamine 2000 (Invitrogen) in 250 μl of Opti-MEM with 10-min incubation], incubated for 20 min, and added dropwise into the Huh7.5 cell cultures that had been preincubated in 2 ml of Opti-MEM for 20 min. The transfected cultures were left for ~16 h and then were subcultured every 2-3 days; the supernatant was collected, filtered (pore size 0.45 μm), and stored at -80°C. To passage virus, Huh7.5 cells grown in six-well plates were incubated with 1 mL transfection collected culture supernatant for ~16 h and then were subcultured every 2-3 days.

### 2.11 qRT-PCR

Total RNA was extracted with TRIzol (DP424, TIANGEN) and reverse-transcribed into cDNA using a reverse transcription kit (R323-01, Vazyme). For qRT-PCR, cDNAs were combined with SYBR master mix (Q311-02, Vazyme). qRT-PCR was performed in triplicate with a Bio-Rad CFX Thermocycler. The data were collected and analyzed with Bio-Rad real-time PCR detection systems and software. The primers are described in **Supplementary 1**.

### 2.12 Western blot

The cells were lysed with lysis buffer (Biosharp, BL504A) in the presence of protease inhibitor cocktail (87786, Thermo Fisher) according to the manufacturer’s instruction. Total protein was dissolved in loading buffer (0.2 M Tris-HCl [PH 6.5], 0.4 M dithiothreitol, 277 mM sodium dodecyl sulfate [SDS], 6 mM bromophenol blue, and 4.3 M glycerol), separated by a 12% SDS-polyacrylamide gel electrophoresis (PAGE) gel, and transferred to immune-blot NC Membranes (1620115, Bio-Rad). The membrane was blocked with 5% skim milk powder in Tris-buffered saline and Tween-20 (TBST) for 1 h and probed with corresponding antibodies. The signal was detected with ECL Western Blotting Substrate (BL520B, Biosharp). Antibodies are described in **Supplementary 1**.

### 2.13 ELISA determination of IL-1β, IL-6 and TNF-α

IL-1β, IL-6 and TNF-α were determined in cell supernatant. Cell supernatant was collected and filtered by 0.45 mm strainer. Cell Extraction Buffer PTR (Abcam) containing a protease inhibitor cocktail (Roche, Switzerland). Human IL-1β (ab214025, Abcam), human IL-6 (ab178013, Abcam), and human TNF-α (ab181421, Abcam) ELISA kits were performed according to the manufacturer’s instructions.

### 2.14 Statistical analysis

Unpaired *t*-test was used to detect the difference between two groups. And comparison among more than two groups of qRT-PCR and ELISA results was assessed by the analysis of *t*-test using the statistical software GraphPad Prism and *P* values < 0.05 were considered statistically significant. Statistical significance is defined as * *P* < 0.05, ** *P* < 0.01, *** *P* < 0.001, or NS (not significant).

## 3 Result

### 3.1 Identification of DEGs and common DEGs among hepatitis C and MASH

**Figure 1:**
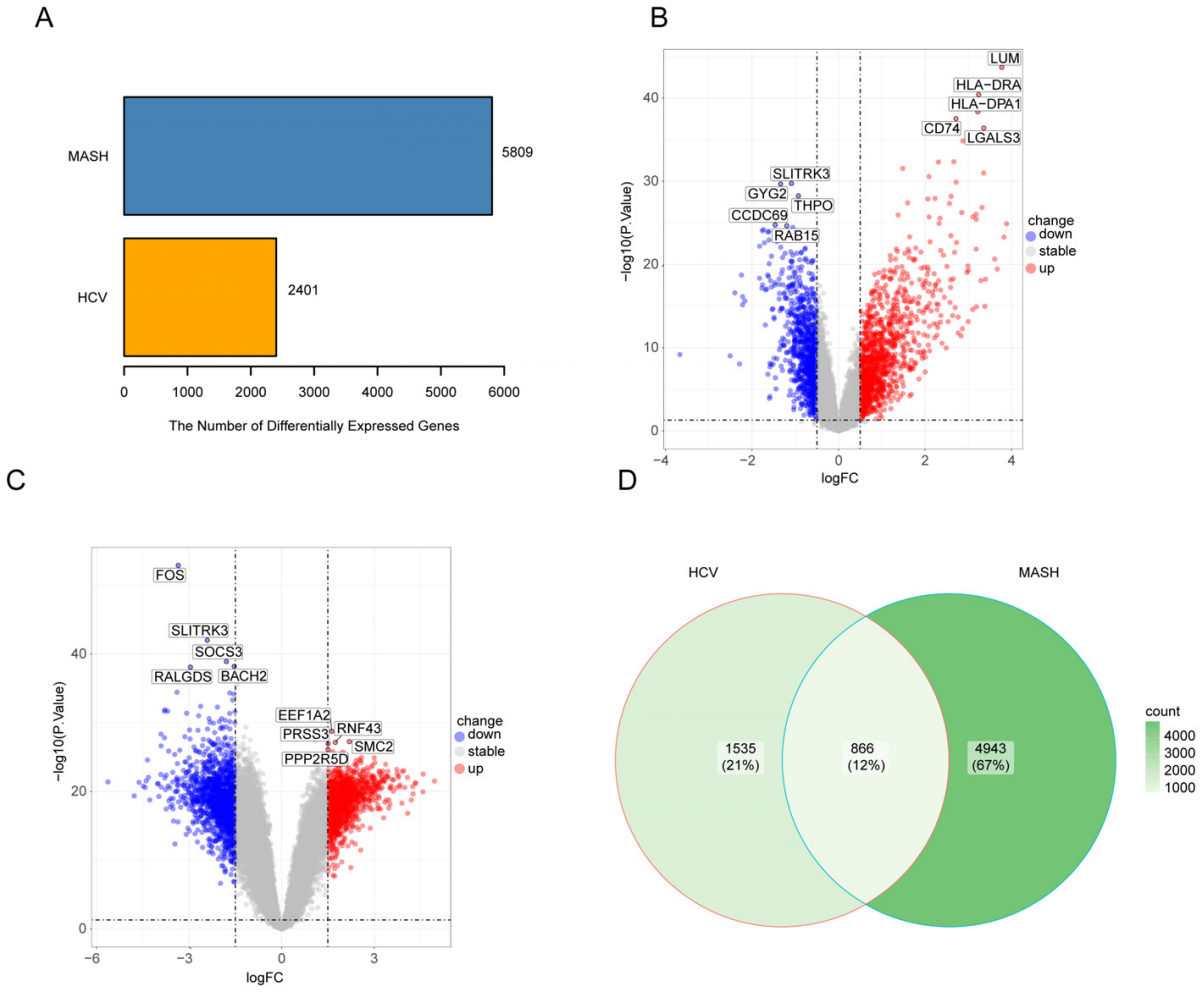
The volcano plot and venn diagram of differentially expressed genes (DEGs). **(A)**, Comparing the number of differentially expressed genes between MASH and HCV; **(B)**, volcano plot of DEGs in GSE14323; **(C)**, volcano plot of DEGs in GSE164760 and GSE89632; **(D)**, Venn diagram showing the overlap of differentially expressed genes between MASH and HCV.

To investigate the interrelationships and implications between MASH and hepatitis C, we analyzed human microarray datasets from the GEO database to identify genes that involved in the development of MASH and hepatitis C. We obtained the datasets GSE164760 and GSE89632 for MASH, and GSE14323 for hepatitis C from the NCBI GEO database. The identification of common DEGs between MASH and hepatitis C suggests a shared molecular pathological basis. In the MASH datasets GSE164760 and GSE89632, a total of 5,809 DEGs were identified. In the HCV dataset GSE14323, a total of 2,401 DEGs were identified (**Figure 1A**). The volcano maps were used to visualize the expression pattern of DEGs in both diseases (**Figures 1B, C**). In addition, the overlap of differential genes between hepatitis C and MASH was evaluated using a Venn diagram analysis. As shown in **Figure 1D**, there were 866 common DEGs between MASH and hepatitis C (**Figure 1D**).

### 3.2 Gene ontology, pathway analysis, and GSEA

**Figure 2:**
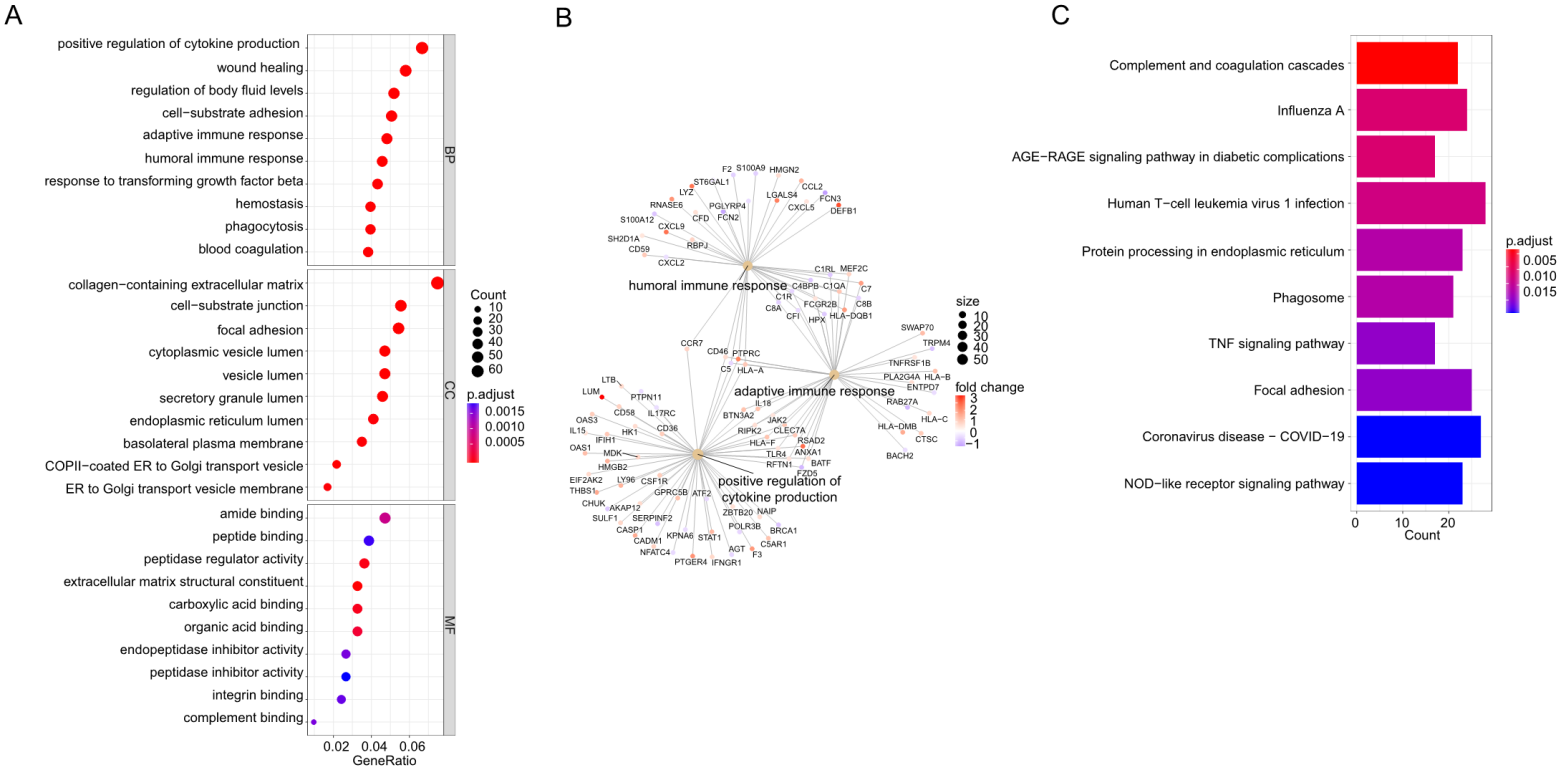
**(A)** Gene Ontology (GO) analysis based on the common differentially expressed genes (DEGs). **(B)** Loop graphs show the correlation between the five most important GO terms and the enriched DEGs. **(C)** Kyoto Encyclopedia of Genes and Genomes (KEGG) analysis based on the common differentially expressed genes (DEGs).

The GO database provides a standardized description of gene products in terms of their function, participating biological pathways, and cellular localization. The results of the GO analysis of hepatitis C or MASH were shown in **Supplementary Figure S1**. In order to further elucidate the biological functions of these differential genes and the signaling pathways involved, GO, KEGG enrichment analyses and GSEA were performed on the above 866 genes to explore the common regulatory pathways. The GO analysis revealed that the common genes might be related to positive regulation of cytokine production, wound healing and regulation of body fluid levels. The analysis of cellular components indicated that the DEGs were mainly associated with the collagen-containing extracellular matrix, cell-substrate junction and focal adhesion. In terms of molecular function, the DEGs were significantly enriched in amide binding, peptide binding and peptidase regulator activity (**Figure 2A**). The Loop graphs show the correlation between the three most important GO terms and the enriched DEGs (**Figure 2B**). These GO terms are humoral immune response, positive regulation of cytokine production and adaptive immune response based on somatic recombination of immune receptors built from immunoglobulin superfamily domains respectively. The results of the KEGG analysis of hepatitis C or MASH were shown in **Supplementary Figure S2**. The KEGG analysis showed that these overlapping DEGs might be primarily involved in complement and coagulation cascades, Influenza A, AGE-RAGE signaling pathway in diabetic complications, Human T-cell leukemia virus 1 infection, Protein processing in endoplasmic reticulum, Phagosome, TNF signaling pathway (**Figure 2C**). More or less, all of these signaling pathways are involved in inflammation. The GSEA was performed for the common DEGs and the result was shown in **Supplementary Figure S3**. The enriched pathway includes cell-substrate junction, endoplasmic reticulum, focal adhesion and regulation of inflammatory response.

### 3.3 PPI network construction and hub genes identification

**Figure 3:**
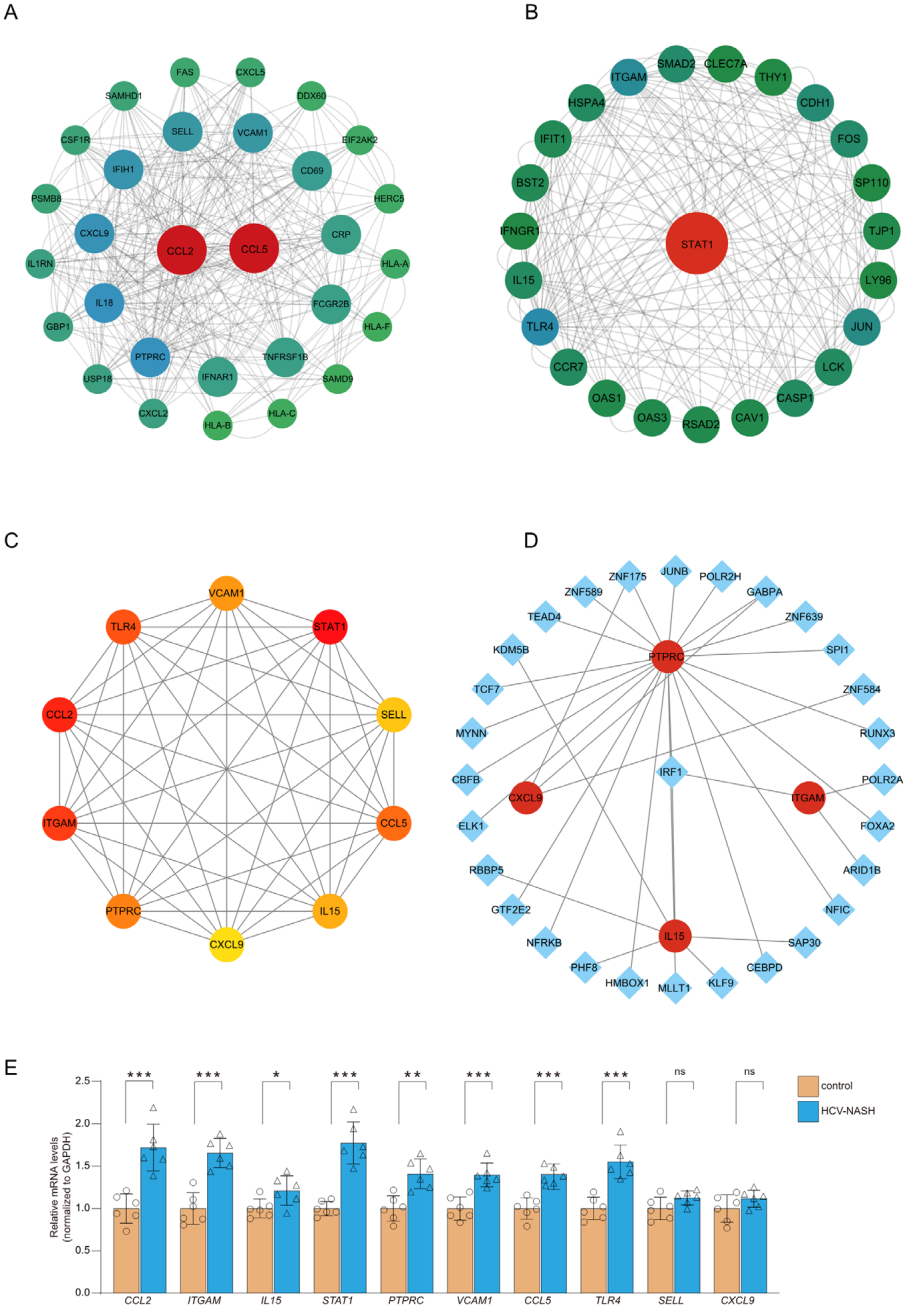
PPI network construction and module analysis. The PPI network of DEGs was constructed in Cytoscape. The most significant module was obtained by MCODE plug-in. **(A)** module score:10; **(B)** module score:8.6. **(C)** PPI network for the top 10 hub genes analyzed by cytoHubba. **(D)** The cohesive regulatory interaction network of DEGs-TFs obtained from the NetworkAnalyst. The square nodes represented TFs, and gene symbols interact with TFs as circle nodes. **(E)** qRT-PCR was performed to detect the levels of hub genes in cell model. Results were represented as mean ± SEM (n = 6, *P < 0.05, **P < 0.01, ***P < 0.001, or NS).

To identify interactions among the common differential genes, a PPI network of DEGs was performed based on the STRING database and the obtained results were imported into Cytoscape for visual analysis. Finally, MCODE plug-in was utilized to identify meaningful gene cluster modules and obtain gene cluster scores (filter criteria: degree cut-off = 2; node score cut-off = 0.2; k-core = 2; max depth = 100). The two modules with the highest score were selected (**Figures 3A, B**). Based on MCC algorithm of CytoHubba plug-in, genes in the top ten were identified as potential hub genes: STAT1, C-C motif ligand 2 (CCL2), ITGAM, PTPRC, CXCL9, IL15, L-selectin (SELL), VCAM1, Toll-like receptor 4 (TLR4), CCL5 (**Figure 3C**, **Table 1**). The qRT- PCR result of 10 hub genes showed, most of the hub genes expression was significantly higher than the control in the cell model. But only the gene expression of *SELL* and *CXCL9* have no significantly between control and hepatitis C-MASH cellular model (**Figure 3E**). Many of them were related to the liver inflammation and hepatocyte injury. Studies from Grohmann M et al. were consistent with a STAT1 gene signature being of functional relevance to the development of MASH (12). Moreover, during the development of chronic viral hepatitis and MASH, CCL2 and CCL5 played an important role (13). In addition, VCAM1 plays a key role in liver inflammation in MASH and hepatocyte TLR4 determine MASH-induced fibrosis (14, 15).

### 3.4 Regulatory network of DEGs-related TFs and miRNAs

**Figure 4:**
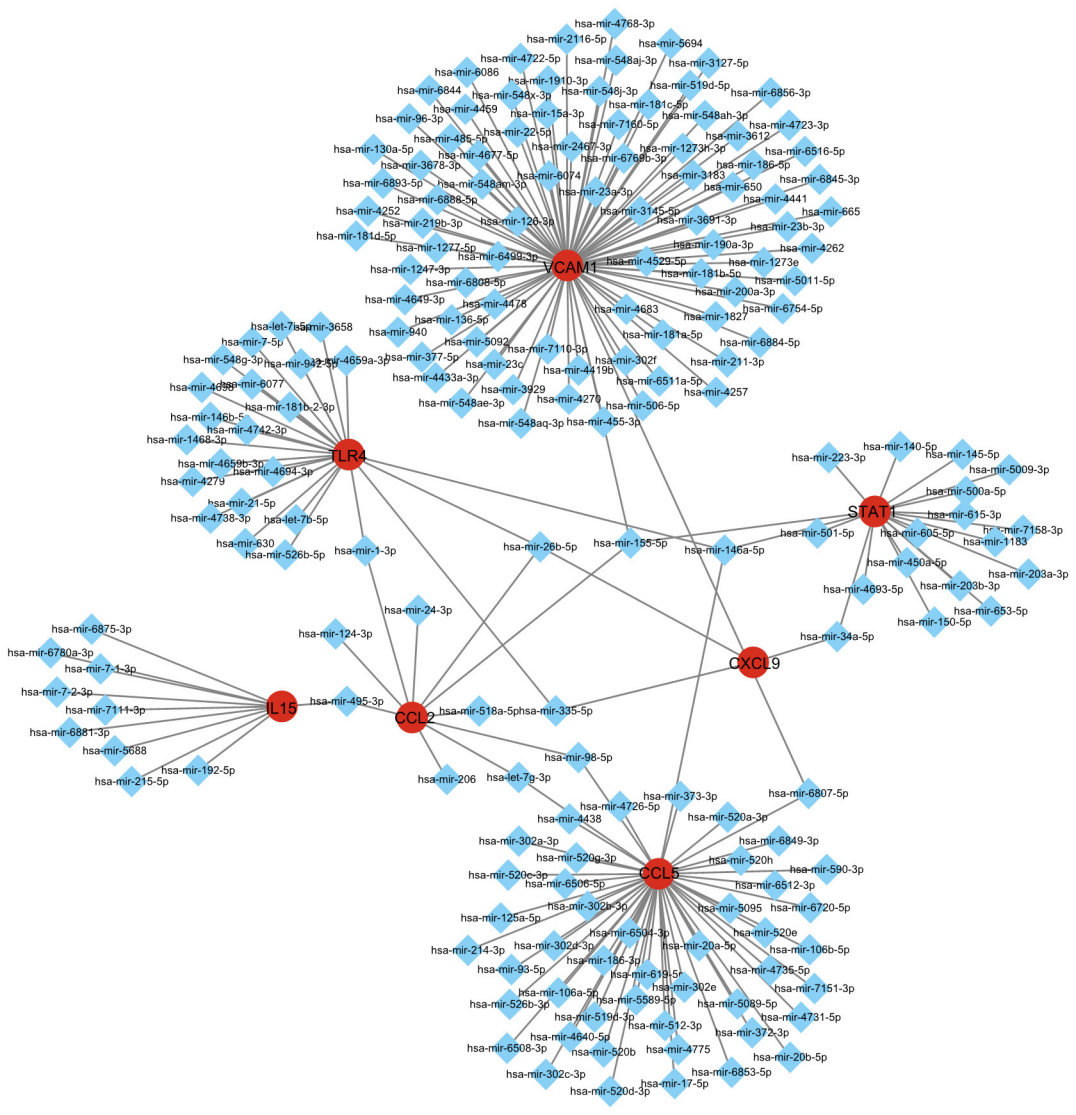
Integrated miRNA-gene interaction networks for the eight hub genes. The square node indicates miRNAs and the gene symbols interact with miRNAs in the shape of a circle.

In order to screen out important regulatory factors of hub genes at the transcriptional level, the Network Analyst platform was used to predict target transcription factors (TFs) and miRNAs of hub genes. And Cytoscape software was utilized to construct interaction network. As shown in **Figure 3D** and **Supplementary Table S1**, Interferon regulatory factor 1 (IRF1) and ZNF175 were the most prominent TF in this network. The most prominent miRNAs were hsa-mir-26b-5p, hsa-mir-146a-5p and hsa-mir-155-5p. hsa-mir-26b-5p was interacted with three hub genes, including TLR4, CXCL9 and CCL2. hsa-mir-146a-5p was interacted with three hub genes, including STAT1, TLR4 and CCL5. hsa-mir-155-5p was interacted with three hub genes, including CCL2, STAT1 and VCAM1 (**Figure 4**, **Supplementary Table S2**). There were studies have shown that miR-26b-5p, miR-155-5p and mir-146a-5p may regulate MASLD by involving in some signal pathway or target gene (16, 17).

### 3.5 Disease-gene network

**Figure 5:**
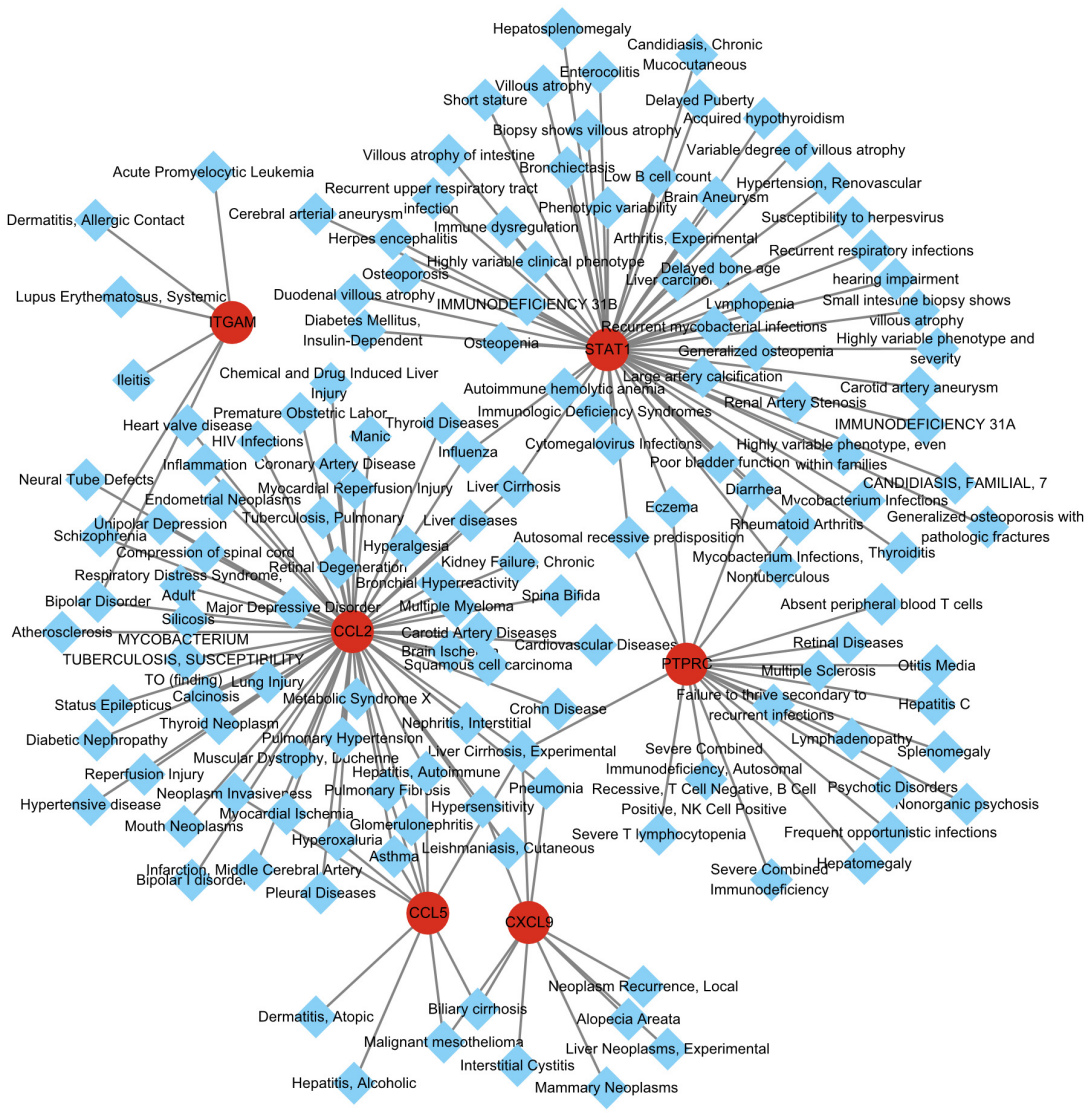
Integrated gene-disease interaction networks for the nine hub genes.

Different diseases may be related to each other, and their regulatory networks usually share at least one or more similar genes. Using the DisGeNET database and the Diseases/Drugs function in Enrichr, we identified diseases and chronic health problems associated with the hub genes. As shown in **Figure 5** and **Supplementary Table S3** illustrates the relationship between hub genes and diseases. Consistent with previous research, Liver Cirrhosis was the most prominent common related diseases of MASH and hepatitis C. HCV-related cirrhosis prevalence increased by 28.7% and MASH-related liver cirrhosis has been shown to be the dominant etiology of cirrhosis, accounting for 59.5% of cases. Overall, cirrhosis accounted for 2.4% of total global deaths in 2017. Suggesting that it is very important to intervene the development of cirrhosis based on these genes (18).

### 3.6 Potential drugs screening and molecular docking

**Figure 6:**
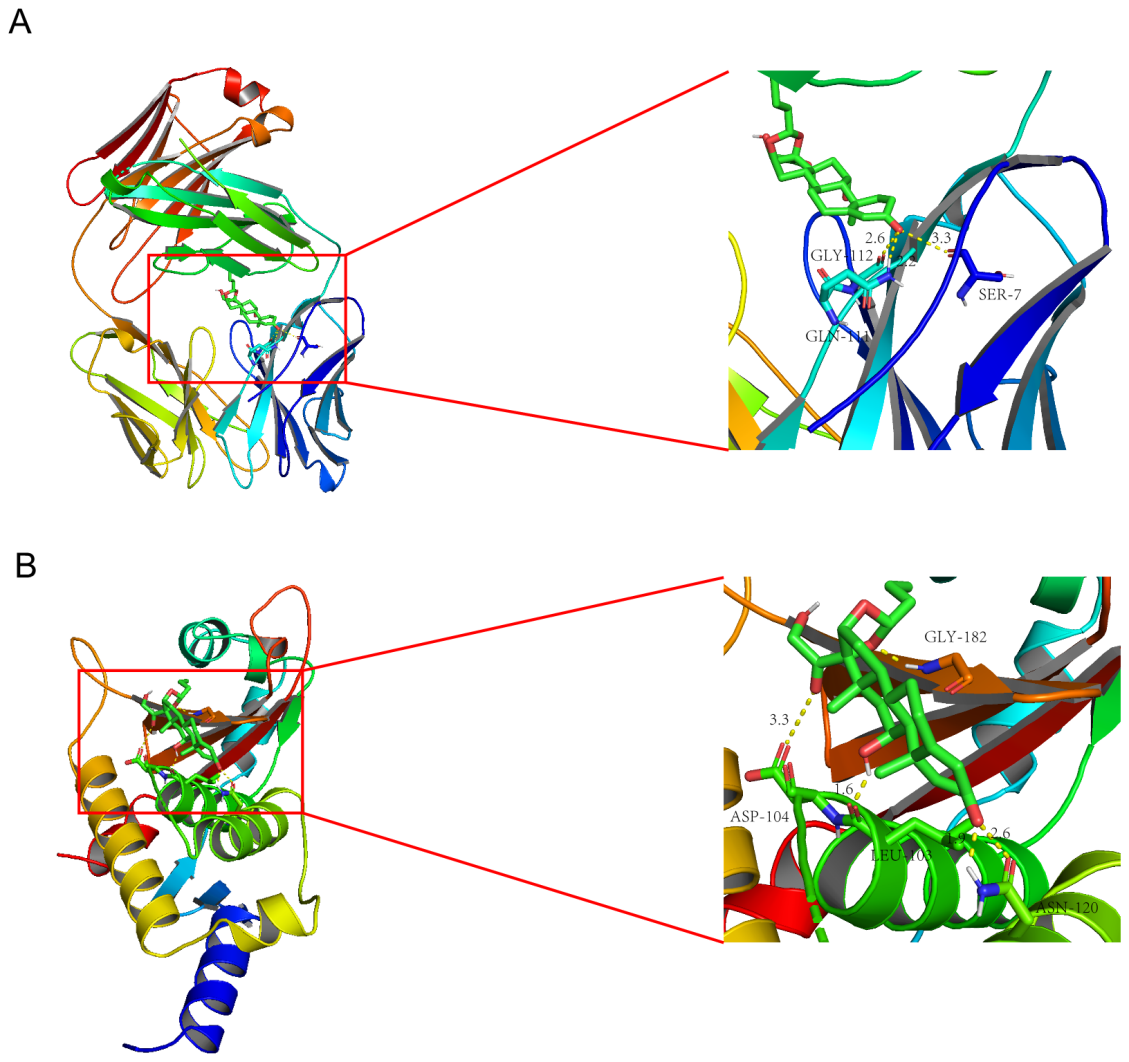
Molecular docking patterns Budesonide **(A)** with the CCL2; Molecular docking patterns Dinoprostone **(B)** with the STAT1.

To explore potential drugs for MASH and hepatitis C, we utilized the Enrichr platform based on the DSigDB database to predict small molecule drugs related to the hub genes. The first 10 potential compounds were finally extracted (**Table 2**). We selected two target proteins (CCL2 and STAT1) with the strongest regulatory interaction with NASH and hepatitis C (**Supplementary Table S3**) for molecular docking analysis to predict their potential therapeutic effects. The docking score between Budesonide and CCL2 was -5.28 (kcal/mol) (**Figure 6A**). The docking score between Dinoprostone and STAT1 was -5.54 (kcal/mol) (**Figure 6B**). Our results provided possible target genes for pharmacological effect of the potential drugs.

**Table 2:** Potential drugs targeting common DEGs among HCV and MASH.

Term	P-value	Adjusted P-value	OddsRatio	Combined Score	Genes
Acetovanillone	1.02E- 11	7.41E-09	511.5641026	12947.91105	ITGAM;VCAM1;STAT1; CCL5;CCL2
glutathione	1.82E- 11	7.41E-09	229.1538462	5667.339993	ITGAM;VCAM1;STAT1; CCL5;CCL2;TLR4
AGN-PC-0JHFVD	1.43E- 10	3.88E-08	160.5810811	3640.504172	PTPRC;VCAM1;SELL;IL15; CCL5;TLR4
budesonide	7.78E-09	0.00000159	282.8794326	5281.678992	VCAM1;STAT1;CCL5;CCL2
dexamethasone	1.64E-08	0.00000245	70.75301205	1268.246275	ITGAM;VCAM1;IL15;CCL5; CCL2;TLR4
Dinoprostone	2.08E-08	0.00000245	103.6596859	1833.578824	VCAM1;STAT1;IL15;CCL5; TLR4
Vanadium pentoxide	0.000000021	0.00000245	217.8032787	3850.285258	CXCL9;VCAM1;STAT1;IL15
Demecolcine	3.23E-08	0.00000329	51.34138857	885.5741104	CXCL9;ITGAM;VCAM1;IL15;CCL5;CCL2;TLR4
Tosyllysylchloromethane	0.000000061	0.00000553	611.5102041	10158.88265	ITGAM;VCAM1;CCL2
PD98059	8.87E-08	0.00000724	76.78210117	1246.796682	ITGAM;VCAM1;STAT1; CCL5;CCL2

### 3.7 Preliminary laboratory validation of the Budesonide in a cell model of MASH and hepatitis C

**Figure 7:**
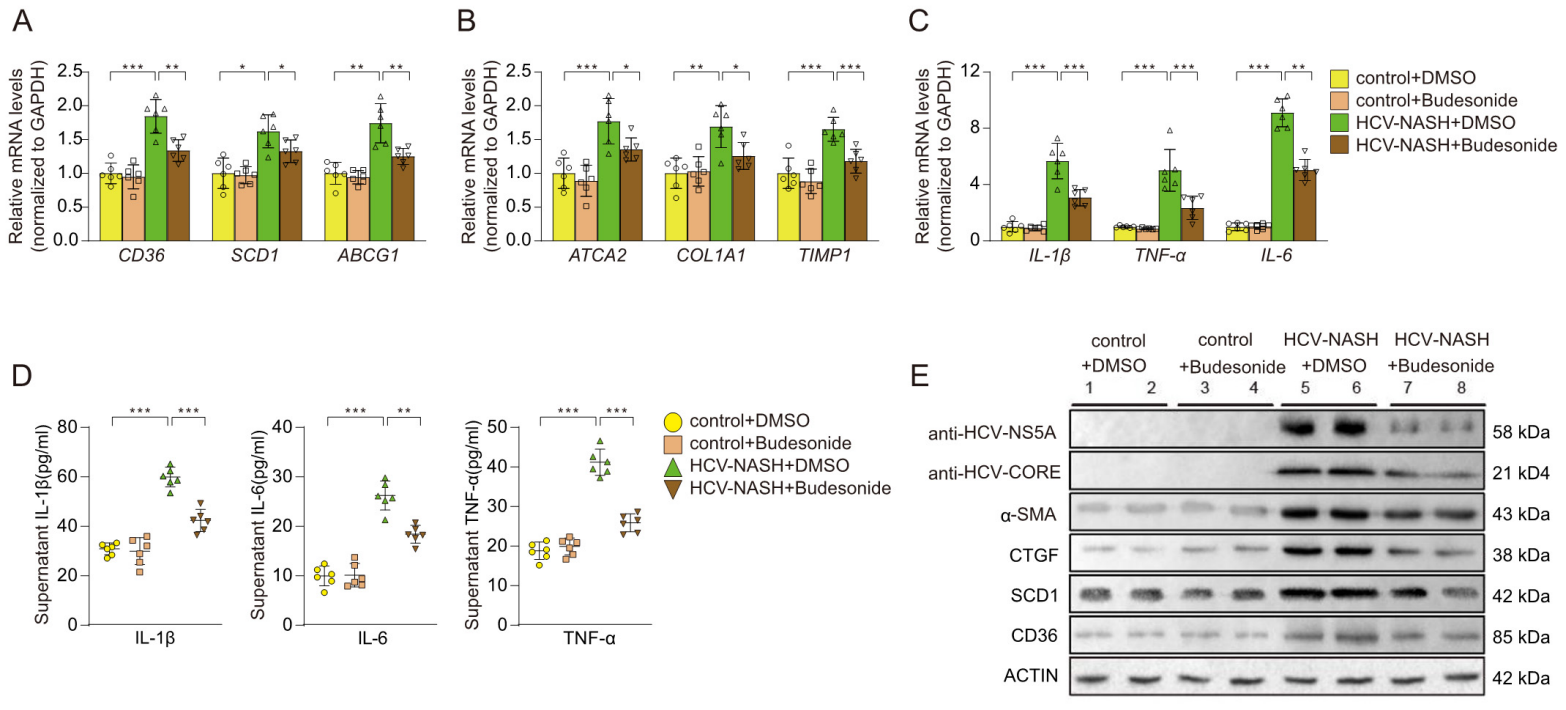
Effects on cellular models after Budesonide treatment. The expression of genes related to glycolipid metabolism **(A)**, fibrosis **(B)**, and inflammatory factors **(C)** were performance by qRT-PCR. The proteins of inflammatory were detected by ELISA **(D)**. **(E)** Immunoblot analysis of the proteins expression, which were HCV and MASH after Budesonide treatment. Data are shown as mean ± SEM. *P < 0.05; **P < 0.01; ***P < 0.001 (*n*=6 per group).

Next, we selected the Budesonide to treat the cellular model of MASH and hepatitis C. The qRT-PCR results showed that Budesonide significantly inhibited the expression of genes related to glycolipid metabolism (**Figure 7A**), fibrosis (**Figure 7B**), and inflammatory factors (**Figure 7C**). The expression of IL-1β, IL-6 and TNF-α were suppressed after Budesonide treatment by ELISA kits (**Figure 7D**). Western blotting results showed that MASH-associated protein (α-SMA, CTGF, SCD1, CD36) and the NS5A, CORE of HCV, expression of which was significantly suppressed after Budesonide treatment (**Figure 7E**). The above results suggest that Budesonide can effectively inhibit the progression of MASH and hepatitis C, at least at the cellular level.

## 4 Discussion

According to the estimation from the World Health Organization (WHO), Hepatitis C is a global epidemic with approximately 58 million people worldwide are chronically infected as of 2019, and 1.5 million people were newly infected globally in 2019 (19). On the other hand, the incidence rate of MASH has been increasing in recent years, and there is a concurrent increase in the prevalence rate of hepatitis C (2). Since liver cirrhosis and HCC are common outcomes of hepatitis C and MASH (20), and HCV infection has a high probability of inducing hepatic steatosis (21), there is considerable interest in understanding the common mechanisms that regulate these two diseases. However, the mechanism of interaction has not been fully understood up to now.

Recently, bioinformatics analysis based on microarray data became a widely used approach to study gene expression profiles in diseases. In this study, we integrated the microarray data of hepatitis C and MASH to explore the common molecular mechanisms underlying these diseases. We identified 866 common DEGs in both diseases and performed GO analysis and KEGG analysis using these genes. The most significantly enriched biological processes was positive regulation of cytokine production, next wound healing, and then regulation of body fluid levels.

Cytokines are chemical messengers in immune system, which consist of chemokines, interferons (IFN), interleukins (IL), tumor necrosis factor (TNF), and colony-stimulating factors (CSF). Cytokines play important roles in host responses to infection, immune responses, inflammation, trauma, sepsis, and cancer. However, aberrant production of cytokines can also contribute to pathologic inflammation. For example, previous studies have shown that the expression of IFN-γ and IFN-γ-inducible chemokines CXCL10, -9, -11 in hepatocytes and lymphocytes of hepatitis C patients increased, which was related to the degree of inflammation (22). Further research found that the increased CRP, IL-1β, and TNF-α were significantly associated with MASH and hepatic fibrosis (23). Recently, a study demonstrated that TNF-α is a key step in Miz1 degradation, resulting in a further reduction in hepatocyte mitophagy, and then promote MASH progression (24). Interestingly, according to a variety of studies, IL-1β, IL-6, TNF-α, and IFN-γ are up-regulated during HCV induced MASH (25). Therefore, the cytokine regulation may be a crucial line between MASH and hepatitis C.

Significantly enriched pathways in KEGG analysis were in order as follows: complement and coagulation cascades, Influenza A, AGE-RAGE signaling pathway in diabetic complications, Human T-cell leukemia virus1 infection, Protein processing in endoplasmic reticulum, Phagosome, TNF signaling pathway, Focal adhesion, Coronavirus disease, and NOD-like receptor signaling pathway. These results indicated that the common pathways between MASH and HCV infection are mainly associated with immunomodulation. It is also suggested that the immune response plays an important role in the development of these two anomalies (26, 27).

After constructing the PPI network of DEGs, we identified the top ten hub genes using the MCC algorithm of CytoHubba plug-in, the top ten were STAT1, CCL2, ITGAM, PTPRC, CXCL9, IL15, SELL, VCAM1, TLR4 and CCL5.

STAT1, belonging to signal transducer and activator of transcription (STAT) family, can be activated by IFN and thereby regulates the gene expressions involved in cell growth, differentiation, apoptosis, and immune response. Cytokine-induced tyrosine and serine phosphorylation facilitate STAT1 homo/hetero- dimerization, nuclear translocation, and transactivation of downstream gene expression. Obesity is one of the triggers of MASH. A recent study reported that the inactivation of negative regulators of the STAT1 signaling in obesity can contribute to the development of MASH and HCC. The oxidation and inactivation of the STAT1 phosphatase TCPTP and heightened STAT1 signaling were evident in MASH in both obese mice and humans. Heightened STAT1 signaling was responsible for the recruitment of activated cytotoxic T cells and the ensuing MASH and fibrosis (12). Meanwhile, another research showed that the expression levels of STAT1 were increased in hepatitis C patients, even higher than that of MASH patients (28). Combined with our analysis, STAT1 could be an important cross-talk gene between MASH and hepatitis C.

CCL2, an important proinflammatory cytokine involved in various inflammatory responses, is also known as monocyte chemoattractant protein 1 (MCP-1). Under the inflammation in the body, monocytes, macrophages, B cells, endothelial cells, and many other cells can secrete CCL2, which plays important roles in rheumatoid arthritis and glomerulonephritis. CCL2 was the first discovered and well investigated C-C chemokine, preferentially binding to its receptor CCR2. Previous studies indicated that the CCL2-CCR2 signaling axis played a role in the promotion of pathological angiogenesis, the survival and invasion of tumor cells, and the recruitment of immune inhibitory cells (29). In a recent study, compared with normal or MASLD patients, plasma CCL2 concentrations were significantly increased in MASH patients. This study showed that a secreted glycoprotein Sparcl1 can promoted the expression of CCL2 in hepatocytes through binding to Toll-like receptor 4 (TLR4) and activation of the nuclear factor kappa B (NF-κB)/p65 signaling pathway (30). It happens that there is a study showed that interaction of HCV core protein with gC1qR could induce CCL2 and CXCL10 secretion in macrophages via NF-κB signaling pathway (31). CCL2 may be a common regulator and novel therapeutic targets in hepatitis C and MASH.

CXCL9, IL15 and CCL5 are also belong to cytokines. A series of studies have shown that CXCL9, IL15 and CCL5 up-regulated significantly in hepatocytes of HCV-infected patients and MASH patients, and existed a correlation between CXCL9 levels and liver fibrosis (32–35). Furthermore, CCL5 could activate hepatic JAK-STAT1/3 and NF-κB signaling and induce hepatocyte damage evidenced (35). Therefore, combined with the results of our GO analysis, it indicated that these cytokines are important factors in chronic liver inflammation, which could be potential therapeutic targets and biomarkers of MASH in the future.

TLR4 is a member of Toll-like receptors family that are of central importance during the host defense against invading pathogens. A growing body of evidence suggests that TLRs, especially TLR4, have a key role in the pathogenesis of chronic inflammatory liver diseases. In Kupffer cells, TLR4 played a critical role in mediating the progression of simple steatosis to MASH by inducing ROS-dependent activation of XBP1 (36). Promoting the degradation of TLR4 can effectively inhibited MASH progression in monkeys (37). Meanwhile, the HCV protein NS5A has the ability to activate the TLR4 gene promoter, thus increasing TLR4 expression. So that the TLR4 expression was higher in the hepatitis C patients group than in the control group (38).

VCAM1 is a member of the immunoglobulin superfamily of cell adhesion molecules and is predominantly expressed on the surface of endothelial cells. It plays a role in firm adhesion of leukocytes to the endothelium. The expression of VCAM1 can be triggered by inflammatory signals. A series of studies reported that VCAM1 is upregulated in murine and human MASH (14, 39). VCAM1 inhibition attenuated proinflammatory monocyte hepatic infiltration, and thereby alleviated liver fibrosis in diet-induced murine MASH models (40). HCV infection also results in upregulation of VCAM1, which are associated with advanced liver fibrosis. Therefore, VCAM1 could be a common liver-fibrosis-enhancing factor in MASH and hepatitis C.

ITGAM, PTPRC and SELL, all belong to cluster of differentiation (CD) antigens, are cell surface molecules expressed on leukocytes and other cells associated with the immune system. ITGAM (antigen-like family member B, CD11b) is an integrin that mediate macrophage adhesion, migration, chemotaxis, and accumulation during inflammation (41–43). PTPRC (CD45) is a leucocyte common antigen and a transmembrane glycoprotein expressed on almost all hematopoietic cells except for mature erythrocytes. SELL (CD62L) is a type-I transmembrane glycoprotein and cell adhesion molecule expressed on most circulating leukocytes. Further studies are urgently needed to explore the roles and detailed anti-inflammatory mechanisms.

We also anticipate target TFs and miRNAs of hub genes so as to find out important regulatory factors at transcriptional level in MASH and hepatitis C. IRF1 is the most prominent TFs. The study found that it interacted with three hub genes, including PTPRC, ITGAM and IL15. And IRF1 is a master transcription factor in the Interferon-γ pathway, playing important roles in apoptosis, inflammation, cell growth and polarization, oncogenesis, and cancers are well documented (44). The most prominent miRNAs, hsa-mir-26b-5p, hsa-mir-146a-5p and hsa-mir-155-5p were found in our study. hsa-miR-155-5p is a crucial regulator that controls cellular pro-inflammatory activities and has been implicated in both HCC and hepatitis C (45). hsa-mir-146a-5p was significantly upregulated in tissues with chronic nasopharyngitis and can target various molecules involved in the NF-κB/NLRP3 pathways (46–48). Thus, targeting these TFs and miRNAs may shed light on the treatment of MASH and hepatitis C.

Besides, we anticipate the connection of common DEGs with different disorder by constructing a hub genes-disease network. As we know that both hepatitis C and MASH cause persistent liver cell damage, which leads to liver fibrosis (2). Consistent with previous research, our results showed that liver cirrhosis was the most prominent in the visible disease network.

We further predicted potential drugs related to the hub genes in patients with MASH and hepatitis C. The results of molecular docking showed a good binding activity between the two most important components (Budesonide and Dinoprostone) and the two important target proteins (CCL2 and STAT1), and the main forms of interaction between components and targets are electrostatic and van der Waals force.

Budesonide is an orally active, second-generation corticosteroid with high anti-inflammatory effect. It has been widely used in the treatment of asthma, pneumonia, ulcerative colitis and other inflammatory diseases (49). Although Budesonide has not been used for the treatment of hepatitis C or MASH, glucocorticoids has strong anti-inflammatory properties through both genomic and non-genomic effects, are widely used in a variety of liver diseases with overactive immune and inflammatory responses, such as liver failure, autoimmune hepatitis and alcoholic liver disease (50). Therefore, we chose budesonide for further experimental verification. The results showed that budesonide significantly suppressed the expression levels of genes related to glycolipid metabolism, fibrosis, and inflammatory factors in cellular model of MASH and hepatitis C. Recent studies found that glucocorticoids could prevent immunologic injury in hepatocytes infected with HBV and might be effective in the treatment of HBV-ACLF (51). A clinical study showed that glucocorticoid can promote the expression of suppressor of cytokine signaling (SOCS) 1 and inhibit the expression level of TNFα and IL-6 in the serum of Acute-on-chronic hepatitis B liver failure (ACHBLF) patients (52). The results were similar to those of our cell experiments. Therefore, Budesonide may be a potential drug for the treatment of MASH and hepatitis C, which also confirms the reliability of the results of our analysis.

Our study has some limitations that need further in-depth work. The bioinformatics analyses were conducted based on one HCV cohort and two MASH cohorts. However, it should be noted that using different datasets would lead to different conclusions, and increasing the number of cohorts or using larger datasets will yield more robust results. Besides, all our findings are validated in cell experiments, while complementary validation using animal experiments is still needed in further studies. Importantly, to enhance the clinical relevance of our study, future efforts should focus on collecting and testing high-quality cohorts of patients with co-existing MASH and hepatitis C. Furthermore, the effects of the identified drugs should be evaluated to confirm the feasibility of our research method between different diseases, such as MASH and hepatitis C.

In conclusion, this study is the first time to use bioinformatics tools to explore the close genetic relationship between MASH and hepatitis C. We identified common DEGs and elucidated the common molecular basis, which predicts multiple common pathways closely related to the two diseases. Our study identified 10 hub genes and their common TFs and miRNAs, which may have a critical influence on the pathophysiological mechanism of hepatitis C and MASH. Next, further experiments will be needed to validate these findings.

## Data Availability

The original contributions presented in the study are included in the article/**Supplementary Material**. Further inquiries can be directed to the corresponding author.
