# Supplementary material for: Common molecular basis for MASH and hepatitis C revealed via systems biology approach
Source: Front Oncol. 2024 Nov 4;14:1442221. doi: 10.3389/fonc.2024.1442221 (PMC11599856; doi:10.3389/fonc.2024.1442221)
Supplement: Supplementary file 1 [file DataSheet1.docx]

Supplementary Material


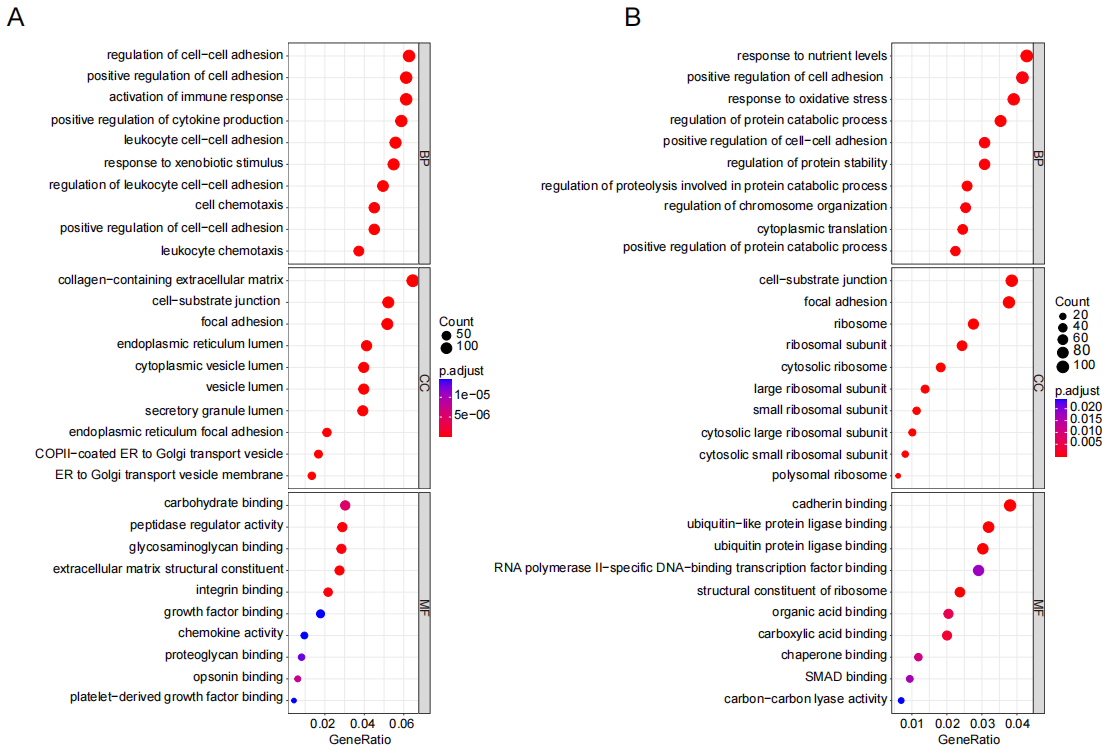


**Supplementary Figure 1.** Bubble graphs indicate the results for (A) Gene Ontology (GO) enrichment analysis in GSE14323. (B) Gene Ontology (GO) enrichment analysis in GSE164760 and GSE89632.


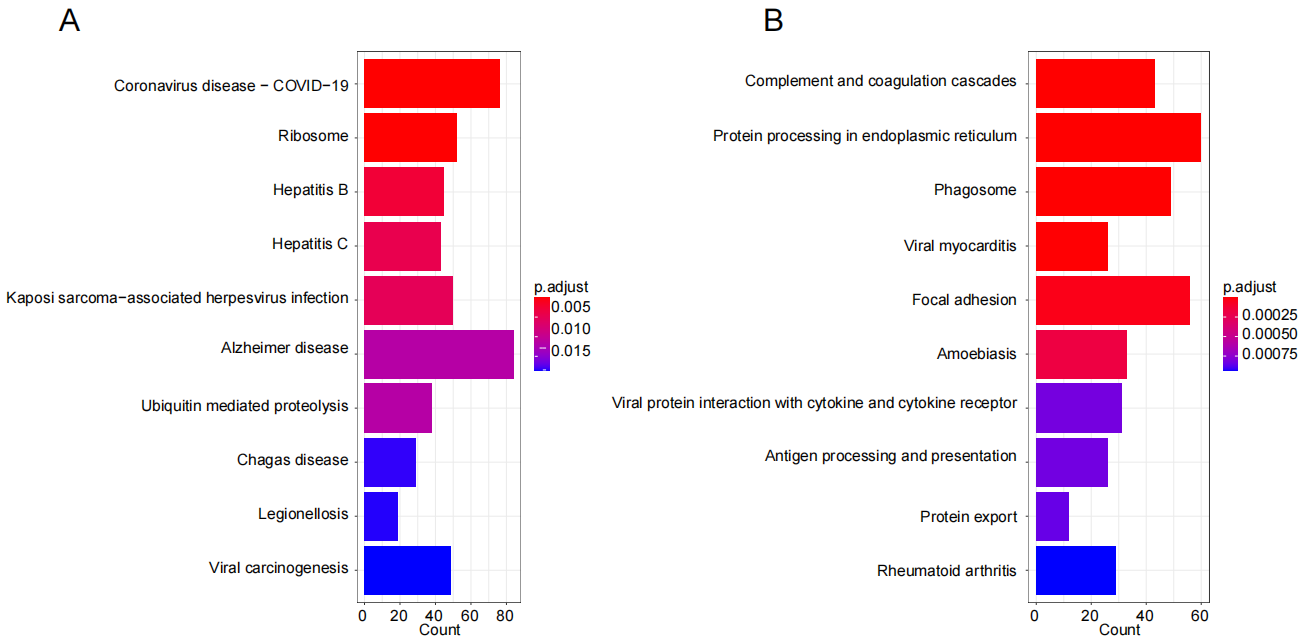


**Supplementary Figure 2.** Kyoto Encyclopedia of Genes and Genomes (KEGG) analysis based on in GSE14323(A) , GSE164760 and GSE89632 (B).


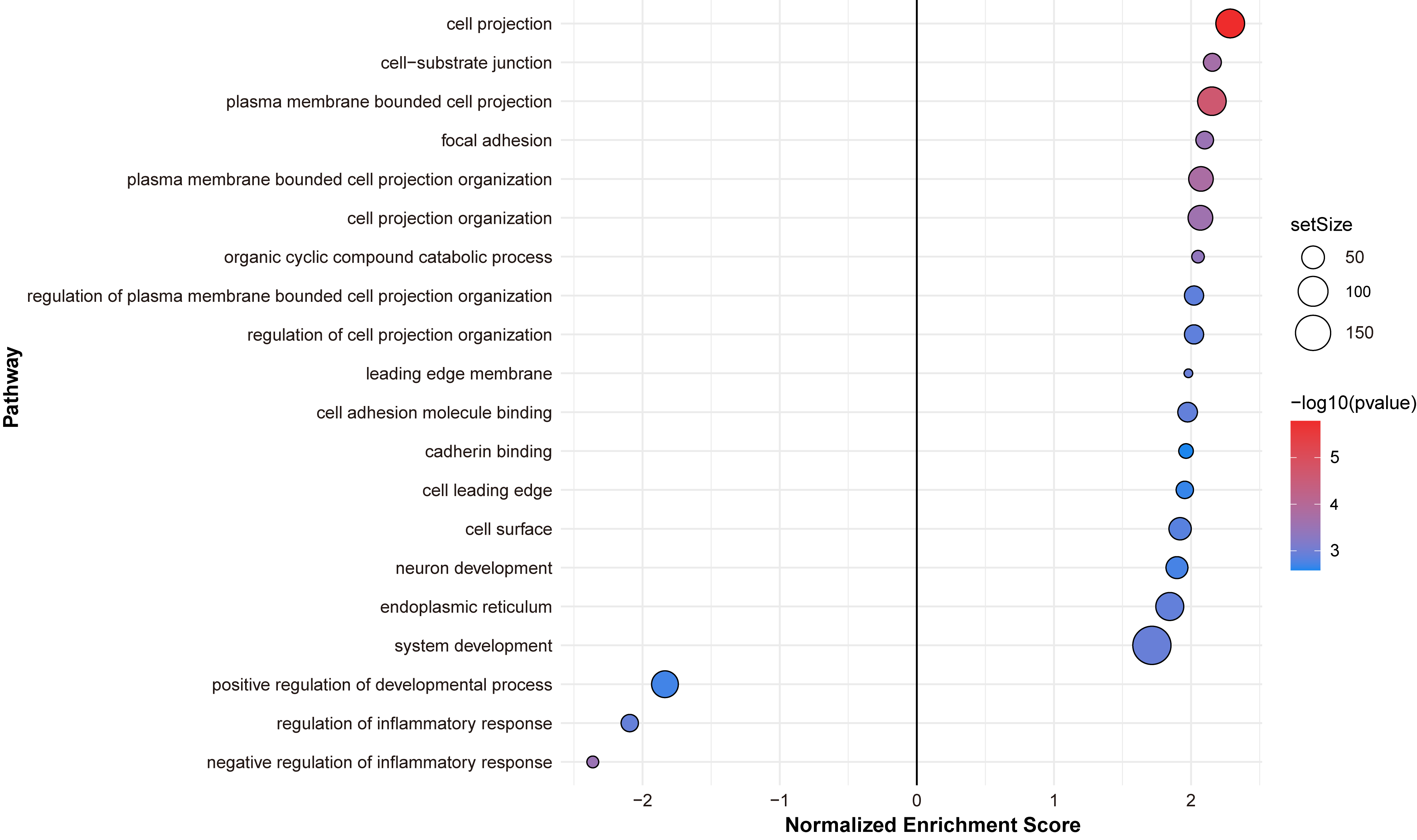


**Supplementary Figure 3.** GSEA based on the differential expressed genes derived from the GSE14323, GSE89632, and GSE164760 datasets. Top 20 enriched pathways were shown.

Table S1 The TFs and their interactions with DEGs

| **Lable** | **Degree** | **Betweenness** |
| --- | --- | --- |
| PTPRC | 21 | 445.5 |
| IL15 | 6 | 145 |
| CXCL9 | 3 | 31.5 |
| ITGAM | 3 | 61 |
| IRF1 | 3 | 225 |
| GABPA | 2 | 29 |
| ZNF175 | 2 | 29 |

| **Label** | **Degree** | **Betweenness** |
| --- | --- | --- |
| STAT1 | 1307 | 999930 |
| VCAM1 | 38 | 33133.91 |
| CCL2 | 27 | 31790.08 |
| CCL5 | 26 | 21100.82 |
| IL15 | 16 | 14076.29 |
| PTPRC | 16 | 15103.21 |
| TLR4 | 13 | 8653.743 |
| CXCL9 | 13 | 13963.92 |
| SELL | 11 | 11696.13 |
| RELA | 5 | 13051.07 |
| SPI1 | 5 | 697.5084 |
| NFKB1 | 4 | 360.1313 |
| JUN | 4 | 10555.4 |
| NFKB2 | 3 | 211.1123 |
| STAT2 | 3 | 99.02107 |
| hsa-miR-145 | 3 | 8617.3 |
| hsa-miR-944 | 3 | 489.9319 |
| ITGAM | 3 | 39.94063 |

Table S2 The microRNAs and their interactions with DEGs

| **Lable** | **Degree** | **Betweenness** |
| --- | --- | --- |
| CCL2 | 61 | 7561.057 |
| STAT1 | 56 | 6327.433 |
| PTPRC | 20 | 2461.21 |
| CCL5 | 11 | 555.7714 |
| CXCL9 | 10 | 843.0286 |
| ITGAM | 6 | 582.5 |
| Liver Cirrhosis Experimental | 4 | 1802.638 |
| Bipolar Disorder | 2 | 355 |
| Schizophrenia | 2 | 355 |
| Hypersensitivity | 2 | 202.0857 |
| Biliary cirrhosis | 2 | 12.6 |
| Pneumonia | 2 | 202.0857 |
| Malignant mesothelioma | 2 | 12.6 |
| Rheumatoid Arthritis | 2 | 265.2857 |
| Diarrhea | 2 | 265.2857 |
| Eczema | 2 | 265.2857 |
| Autosomal recessive predisposition | 2 | 265.2857 |

Table S3 Regulatory interactions between common DEGs and diseases
